# Warning of severe pulmonary embolism after cerebral angiography: A case report and literature review

**DOI:** 10.1097/MD.0000000000039635

**Published:** 2024-09-27

**Authors:** Xiaolin Zhang, Wenjing Zhang, Wangfang Yu, Wei Yu, Wei Shen, Qi Wu, Zhiping Huang, Yonghua Zhang

**Affiliations:** a Department of Neurosurgery, The Ningbo Beilun District People’s Hospital, Ningbo, China; b Department of Anesthesia, The Ningbo Beilun District People’s Hospital, Ningbo, China; c Department of Cardiology, The Ningbo Beilun District People’s Hospital, Ningbo, China; d Department of Critical Care Medicine, The Ningbo Beilun District People’s Hospital, Ningbo, China; e Department of Respiratory, The Ningbo Beilun District People’s Hospital, Ningbo, China.

**Keywords:** case report, cerebral angiography, cerebrovascular disease, pulmonary embolism, VTE, warning

## Abstract

**Rationale:**

Acute pulmonary embolism (PE), which can lead to cardiac and respiratory arrest, is a rare complication of cerebral angiography. However, neurologists do not pay attention to this.

**Patient concerns:**

A 47-year-old male with a history of type 2 diabetes was admitted to our hospital for evaluation of surgical indications for unruptured ophthalmic aneurysms. After cerebral angiography, a fatal PE occurred. Through rapid identification and effective drug treatment, the patient recovered and was discharged.

**Diagnoses:**

A diagnosis of fatal PE was made based on the bedside ultrasonography and blood d-dimer level.

**Interventions:**

Cardiopulmonary resuscitation and intravenous thrombolysis of “50 mg alteplase” for continuous intravenous drip for 2 hours.

**Outcomes:**

The patient was recovered and no special discomfort was reported.

**Lessons:**

PE is a rare complication of cerebral angiography, but the fatality rate is very high. Neurologists must not only early identify and effectively treat this complication, but more importantly, pay attention to this complication, prevent it in advance, and reduce the occurrence of catastrophic events.

## 1. Introduction

Cerebrovascular disease is a major disease that threatens human health and has 5 characteristics: high prevalence, high disability, high mortality, high recurrence, and high treatment costs.^[1]^ At present, cerebral angiography is the “gold standard” for the diagnosis of cerebrovascular diseases, and it is an important examination to determine both the types of cerebrovascular diseases and the surgical plan.^[2]^ With the popularization of neurointerventional technology, increasing number of hospitals are skillfully using cerebral angiography technology to diagnose cerebrovascular diseases. Acute pulmonary embolism (PE), which can lead to cardiac and respiratory arrest, is a rare complication of cerebral angiography.^[3]^ One patient presented with severe acute PE in more than 2000 cases of cerebral angiography at our hospital. After early identification, cardiopulmonary resuscitation, timely thrombolysis, and other treatment, the patient was completely cured. The diagnosis and treatment process of this patient were analyzed retrospectively, and the high-risk factors, early identification methods, treatment strategies, and preventive measures were analyzed in combination with the literature to provide a warning to neurologists.

## 2. Case report

**Figure 1.**
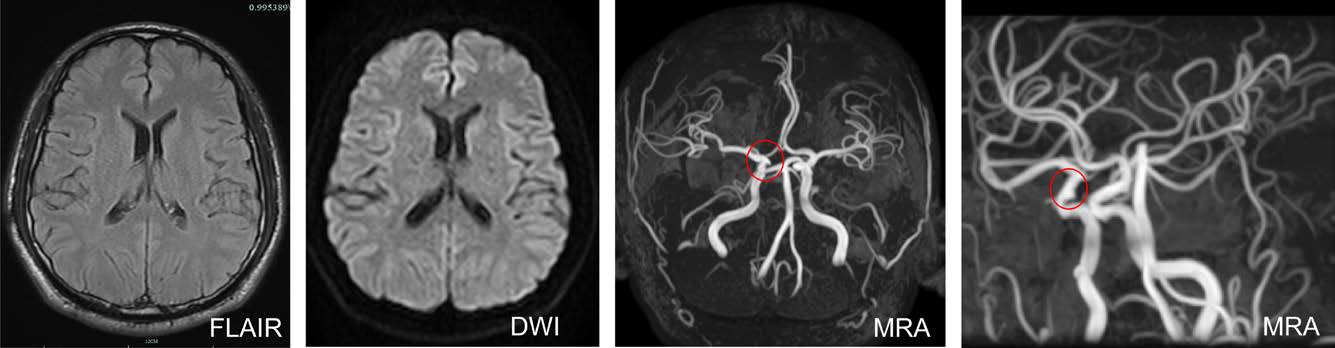
MRI Flair and DWI sequence is normal, and MRA showing an aneurysm (red circle) located at the ophthalmic segment of the right internal carotid artery. MRA = MR angiography, MRI = magnetic resonance imaging.

**Figure 2.**
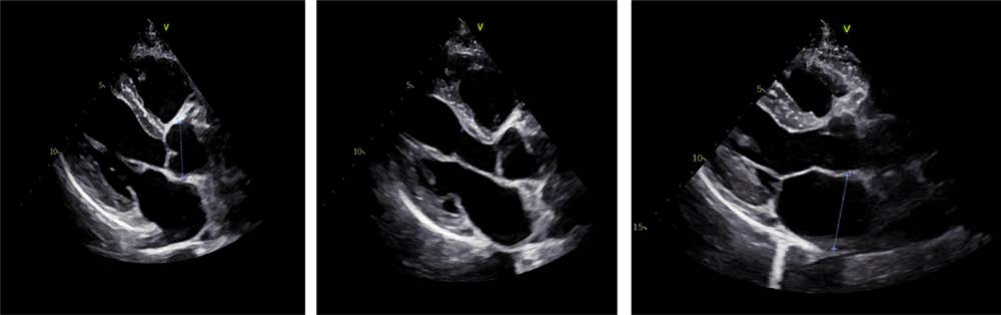
Cardiac ultrasound: cardiac measurements: AO: 32 mm, IVSd: 9 mm, LVDd: 46 mm, LVFS: 41%, LA: 41 mm, LVPWd: 8 mm, LVDs: 27 mm, LVEF: 72%. The left atrium is full, tricuspid regurgitation is slight, and pericardial cavity has few hydrops.

**Figure 3.**
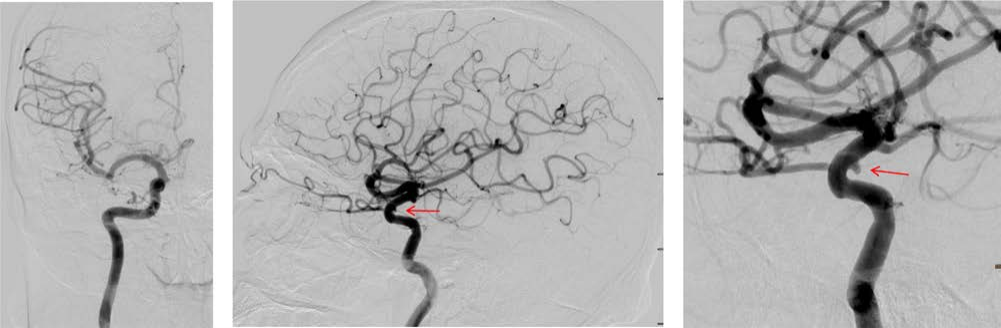
Cerebral angiography demonstrates an aneurysm (arrow) located at the ophthalmic segment of the right internal carotid artery.

In June 2023, a 47-year-old Chinese man with a history of type 2 diabetes was admitted for “dizziness for 4 days and unconsciousness for 30 minutes.” Before admission, cervical computed tomography (CT) showed “C3/4,C4/5,C5/6,C6/7 disc herniation (central type)” and MRI + MRA showed “right internal carotid artery ophthalmic aneurysm” (Fig. 1). This time, hypoglycemia and unconsciousness were caused by failure to eat in a timely manner after insulin injection, which lasted for 30 minutes. After oral glucose supplementation, the symptoms disappeared and the patient was admitted to the hospital. Blood glucose levels were controlled under treatment, and blood indicators including blood routine, blood coagulation function, liver and kidney function, and electrocardiogram gradually improved. Cardiac color Doppler showed atrial fullness, mild tricuspid regurgitation, and a small effusion in the pericardial cavity (Fig. 2). Deep venous thrombosis (DVT) of both lower limbs could be seen in the bilateral femoral vein, popliteal vein, anterior tibial vein, and posterior tibial vein. The blood flow in the great saphenous vein was unobstructed. On the 3rd day after admission, the Seldinger technique was applied to puncture the femoral artery under local anesthesia, and cerebral angiography revealed an ophthalmic aneurysm of the right internal carotid artery (Fig. 3). After the operation, the right lower limb was immobilized for 24 hours and the puncture point was compressed with sandbags for 6 hours. Since the cerebral aneurysm was small and regular, it was a dynamic recovery examination and observation, and no surgery was needed for the time being. The patient was discharged and suddenly fainted when he tried to get out of bed.

**Figure 4.**
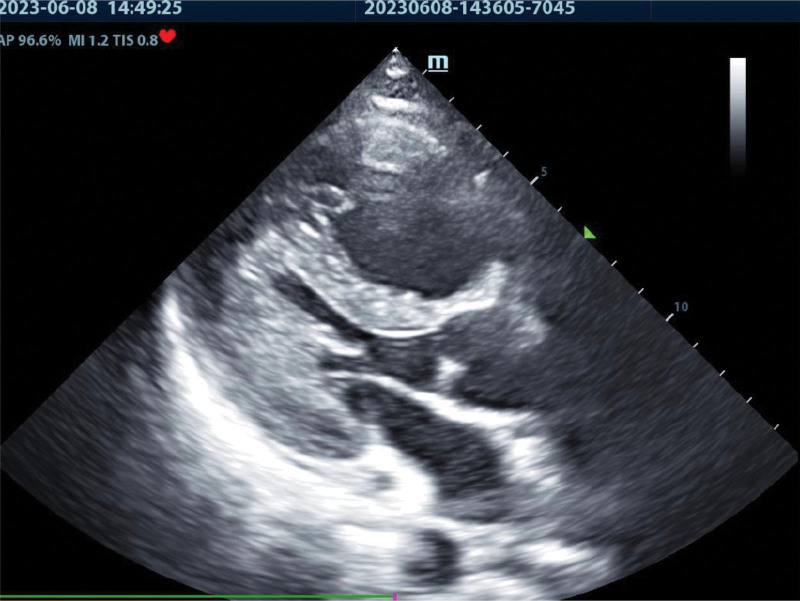
Cardiac ultrasound: the right heart was enlarged, the left heart became smaller, and the cardiac activity was weak, which was consistent with the ultrasonic changes of pulmonary embolism.

**Figure 5.**
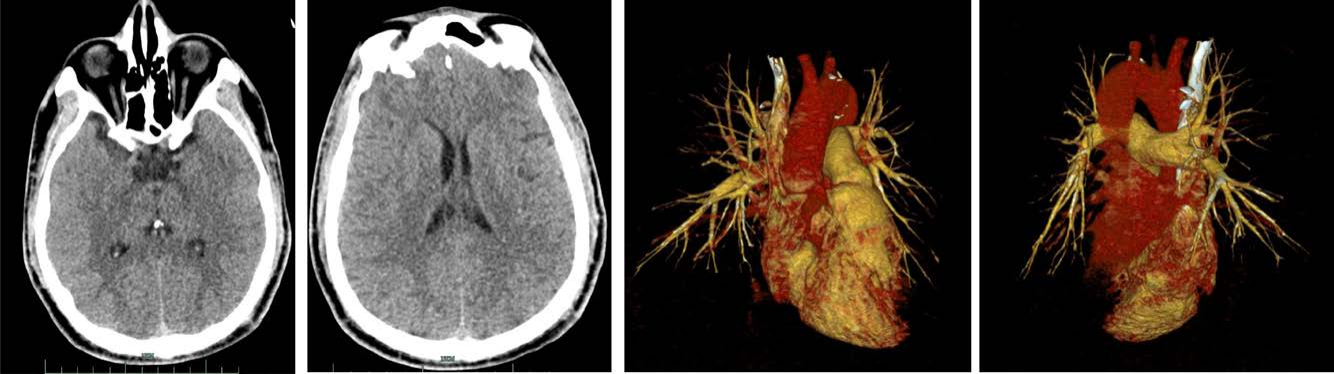
Craniocerebral CT: cerebral tissue has no obvious signs of hemorrhage and infarction. Pulmonary artery CTA: the main trunk and branches of both superior and inferior pulmonary arteries were well displayed, running naturally, no obvious dilatation and stenosis, and no obvious filling defect in the official cavity. The position of the heart with the aorta, pulmonary artery, and pulmonary vein was normal. CT = computed tomography, CTA = CT angiography.

Rapid diagnosis of respiratory and cardiac arrest, adult 999, cardiopulmonary resuscitation, tracheal intubation, rapid infusion, and other treatments were initiated. The rescue effect was unsatisfactory, and an acute PE was considered. Bedside deep vein examination of both lower extremities showed no thrombosis, and echocardiography revealed marked enlargement of the right heart, shrinkage of the left ventricular cavity, and weakness of cardiac activity. It was conformed as a PE by ultrasonication, as shown in Figure 4. The emergency blood d-dimer level was 119,408 μg/L, troponin < 0.025ng/mL, and myocardial zymogram CK-MB was 18.61 ng/mL. After thrombolysis, the effect of cardiopulmonary resuscitation was obvious, and spontaneous heart rhythm, blood pressure, and oxygen saturation gradually returned to normal. The patient was transferred to the intensive care unit for further rescue treatment, and a B ultrasound was performed 2 hours after resuscitation, which showed smooth deep venous blood flow in both lower limbs. Brain CT revealed no signs of cerebral hemorrhage or infarction. Pulmonary artery CT angiography (CTA) showed that the trunk and branches of the upper and lower pulmonary arteries on both sides were well displayed, running naturally. No obvious expansion and stenosis were found, no obvious filling defect was found in the official cavity, and the relationship between the heart and aorta, pulmonary artery, and pulmonary vein was normal, as shown in Figure 5.

**Figure 6.**
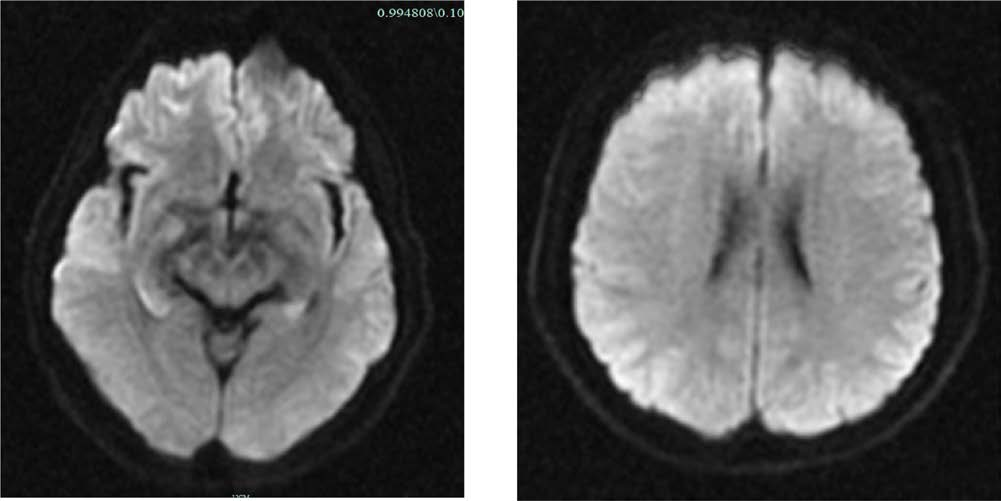
Craniocerebral MRI (DWI): cerebral tissue has no obvious signs of infarction. MRI = magnetic resonance imaging.

On the 2nd day after resuscitation, the right heart was slightly larger on color Doppler ultrasonography. Tricuspid valve is slightly regurgitated, with a small amount of effusion in the pericardial cavity. Doppler ultrasound of the inferior vena cava showed unobstructed blood flow, and no obvious mural thrombi were found. On the 4th day after resuscitation, there was no obvious abnormality in the MRI plain scan and DWI sequence, as shown in Figure 6. On the 7th day after resuscitation, the patient recovered and was discharged. The patient was well at 6-month follow-up.

## 3. Discussion

Venous thromboembolism (VTE) includes DVT and PE, both of which occur in 2 different stages of the same disease. DVT is referred to as thrombosis formed in deep veins, while PE is referred to as arterial embolism of the pulmonary vein contributing to thrombosis breaking off from the deep vein and migration to the pulmonary vein, leading to dysfunction of the pulmonary circulation and respiration. VTE is mainly related to long-term bed rest, which leads to venous blood stasis, hypercoagulability, and vascular wall injury.^[4]^ Acute PE is a common cardiovascular disease and 1 of 3 fatal cardiovascular diseases. According to statistics, 90% of PEs in neurosurgery patients come from DVT of the lower limbs, and its mortality rate could reach 9 to 50%.^[5]^ The warning for this case is as follows:

### 3.1. Pay attention to the risk classification of thrombosis

Caprini Thrombosis Assessment Form was developed by American scholar Caprini. It was used clinically in the 1980s and has become a relatively mature risk-assessment tool since 2010. It assesses more than 30 risk factors and assigns corresponding scores according to the different risk factors. By 2005, a relatively mature risk-assessment model was developed. The scale includes 33 risk factors, such as the general situation, body mass index, and VTE history.^[6]^ According to the various effects of different factors on VTE risk, risk factors were assigned, and the score of each risk factor was 1 to 5. According to the total score, they were divided into 3 groups, with low risk ≤ 2 points, medium risk 3 to 4 points, and ultra high risk ≥ 5 points, which can provide a reference for medical staff to implement different intervention measures according to the degree of risk. Currently, a large number of studies have confirmed that the scale has a wide range of practicality and high effectiveness and can better evaluate the risk of VTE in critically ill patients.^[6–8]^

### 3.2. Monitoring of d-dimer

As a molecular marker of hypercoagulability and hyperfibrinolysis in vivo, d-dimer has high sensitivity in the diagnosis of thromboembolic diseases and can reflect the severity of diseases. In acute thrombosis, coagulation and fibrinolysis are activated simultaneously, which can cause an increase in the plasma d-dimer levels. The negative predictive value of d-dimer detection was very high. Acute PE and DVT can be ruled out when the level is normal, especially in low-risk suspicious patients.^[9,10]^ At the time of admission, the plasma d-dimer was 252 µg/L, and the ultrasound revealed unobstructed blood flow in the deep veins of both lower limbs, and no venous thrombosis or intermuscular venous thrombosis was found. The patient was graded according to the Caprini scale after cerebral angiography, with a score of 4, indicating moderate risk. Cerebral angiography through the femoral artery is an intravascular procedure that carries the risk of vascular wall injury. After the puncture through the femoral artery, long-term pressure dressing and limb immobilization are needed to avoid bleeding at the puncture site, and the patient’s bed rest time increases. Limb immobilization and the effect of the gastrocnemius muscle on the pump of lower-limb blood reflux decreases or disappears, which increases the risk of thrombosis. The patient had diabetes and arteriosclerosis (arterial plaque), which are the most important risk factors for DVT. Thus, the occurrence of acute PE in this patient should consider the location of acute PE caused by thrombus shedding in the right femoral vein, related to bed rest and limb immobilization after angiography; however, it does not rule out its own vascular conditions and coagulation. d-dimer level after respiratory and cardiac arrest (119,408 µg/L) was significantly higher than that before the operation (252 µg/L), indicating a high probability of acute PE.

### 3.3. Color ultrasound examination

Color Doppler ultrasound examination with high sensitivity and accuracy is widely used in medical facilities. It is a priority for the diagnosis of DVT, suitable for screening and monitoring, and its accuracy in the diagnosis of femoral popliteal vein thrombosis is high (>90%).^[11]^ Femoral artery puncture cerebral angiography may accidentally injure the femoral vein, coupled with lower-limb immobilization and sandbag compression at the puncture point, leading to venous thrombosis. The risk of PE is highest in the first few days of thrombosis due to the breakableness of thrombosis and the effect of the fibrinolytic system. As a common technique for diagnosing acute PE in primary medical institutions, echocardiography is convenient for emergency treatment and rescue and can provide direct and indirect signs of acute PE. The direct sign is the discovery of a thrombus in the proximal pulmonary artery or right ventricular cavity. If the clinical manifestations were suspected to be acute PE, the diagnosis could be confirmed, but the positive rate was low. Indirect signs are mostly manifestations of right heart overload, such as a decrease in the local motion amplitude of the right ventricular wall, enlarged right ventricle and/or right atrium, increased tricuspid regurgitation velocity, movement of the ventricular septum to the left, and a widened pulmonary trunk.^[12]^ Previously, when patients without pulmonary vascular diseases were diagnosed with acute PE, the right ventricular wall was generally not thickened, and the systolic pressure of the pulmonary artery rarely exceeded 35~40 mm Hg. Therefore, it is helpful to combine the clinical manifestations with the characteristics of echocardiography to distinguish between acute and chronic PE.

### 3.4. CT pulmonary angiography and pulmonary angiography

With a sensitivity of 83% and a specificity of 78 to 100%,^[13]^ CT pulmonary angiography is acknowledged as an important noninvasive examination technique for the diagnosis of acute PE. As the “gold standard” for the diagnosis of acute PE, pulmonary angiography is feasible when other tests failed to confirm the diagnosis, and contraindications of pulmonary angiography were not detected. For patients with hemodynamic instability who are suspected of having ACS and are sent directly to the catheter room, pulmonary angiography and percutaneous catheter intervention can be considered after ACS is ruled out.^[14]^

### 3.5. Pay attention to early identification

No blood thrombosis was identified on the Color Doppler ultrasound of both lower extremity veins when the patient was admitted to the hospital, and the grade on the Caprini Scale was low. After cerebral angiography, the Caprini Scale grade of the patient changed from low to high risk, and the femoral vein may have been accidentally injured by a femoral artery puncture. This patient was in a high-risk group for arteriosclerosis and diabetes. After surgery, the patient’s right lower limb was braked, and the puncture point was pressed using a sandbag, which easily led to the formation of deep vein thrombosis in the lower limbs. Before releasing the right lower limb and puncture point, failure to monitor plasma d-dimer and ultrasonic screening of DVT in the lower limbs led to a missed diagnosis of DVT in the lower limbs. After the patient got out of bed, the DVT fell off and reached the pulmonary artery of the patient with blood circulation, which led to acute PE, syncope, and cardiac arrest.

### 3.6. Significance of treatment timeliness

In cases of respiratory and cardiac arrest, patients with acute PE should undergo cardiopulmonary resuscitation immediately and be transferred to the rescue unit to start the multidisciplinary meeting of adult 999, tracheal intubation, cardiology, respiratory, critical care medicine, and ultrasound. Once the patient is suspected of acute PE, after acute myocardial infarction and thrombolysis contraindications are ruled out, intravenous thrombolysis of “alteplase” is quickly started, and the dose is 50 mg for continuous intravenous drip for 2 hours.^[15]^ During thrombolysis, it is vital to note the patient’s vital signs, electrocardiogram, coagulation function, and implementation of pulmonary artery CTA to determine the effectiveness of thrombolysis. If the thrombolytic effect is lower than expected, interventional surgery for acute PE is an alternative.^[16]^ Patients with successful thrombolysis need to take anticoagulants for 3 months postoperatively to prevent recurrence.

### 3.7. Pay attention to health education

Puncture arterial bleeding and subcutaneous hematoma are common complications of postcerebral angiography. To avoid these complications, education should be provided according to the method of arterial hemostasis. After the routine operation, the punctured side limb was bebraked and bedridden for 24 hours, the puncture point was bandaged for 24 hours, and the sandbag was pressed for 6 hours. There are some high-risk factors for VTE, such as long-term bed rest and limb immobilization, middle or elderly age characteristics, and chronic diseases, including hypertension, hyperlipidemia, diabetes, and arteriosclerosis. This case also reminds the necessity of strengthening the education of ankle pump activities of lower limbs during bedridden periods and breaking after radiography, and also to do a good job of supervising patients and their families to ensure the implementation of preventive measures. Simultaneously, optimization of the surgical method is urgent for radiologists. The use of new hemostatic devices (Perclose Proglide and Angio-Seal VIP) can stop bleeding quickly, reduce postoperative limb braking and bed rest time, and allow patients to get out of bed as soon as possible, reducing the occurrence of deep vein thrombosis.^[17]^ Additionally, the recent rise in radial cerebral angiography has dramatically contributed to the reduction in bed rest length.^[18]^

Acute PE is a rare and serious complication of cerebral angiography. Paying attention to the high-risk group of thrombosis and the risk classification of thrombosis, monitoring d-dimer and deep vein color Doppler ultrasound before and after surgery, and strengthening health education can often avoid the occurrence of acute PE. Echocardiography and pulmonary artery CTA are beneficial for diagnosis, early identification, and timely thrombolysis, eventually improving the treatment outcome of patients with acute PE.

## Author contributions

**Project administration:** Xiaolin Zhang, Wangfang Yu, Wei Shen, Qi Wu, Zhiping Huang, Yonghua Zhang.

**Writing – original draft:** Xiaolin Zhang.

**Data curation:** Wenjing Zhang.

**Conceptualization:** Wei Shen.

**Writing – review & editing:** Wei Yu.
